# Quantum Chaos—Dedicated to Professor Giulio Casati on the Occasion of His 80th Birthday

**DOI:** 10.3390/e25091279

**Published:** 2023-08-31

**Authors:** Marko Robnik

**Affiliations:** Center for Applied Mathematics and Theoretical Physics, University of Maribor, SI-2000 Maribor, Slovenia; robnik@uni-mb.si; Tel.: +38-62-235-5350 (ext. 5351)

Quantum chaos is the study of phenomena in the quantum domain which correspond to classical chaos. More generally, we speak of *wave chaos* (see below). In classically chaotic systems, we observe the sensitive dependence on initial conditions manifested in the *asymptotically* (*for infinite times*) exponential divergence of nearby orbits, with the rate being the largest positive Lyapunov exponent. This is a consequence of the nonlinearity of the underlying classical equations of motion. In quantum mechanics, we study the properties of the solutions of the time-dependent Schrödinger equation of motion, which is linear. Here, we observe, firstly, that the concept of orbits does not exist due to the Heisenberg uncertainty principle, and secondly, that the time-evolution of solutions (in the case of bound systems with a discrete energy spectrum) is always almost periodic due to the linearity. Consequently, an attempt to define *asymptotic* quantum Lyapunov exponents (for infinite times) results in the conclusion that they must be zero. Nevertheless, under certain conditions for *finite* times, the behavior of certain dynamical quantities, such as an out-of-time-ordered correlator (OTOC), can follow an exponential law, thereby mimicking classical behavior, but typically only up to the Heisenberg time (i.e., the ratio of the Planck constant and the mean energy spacing). In this sense, the dynamical chaos in quantum mechanics does not exist. However, there exist stationary aspects of classical chaos, namely the structure of the phase portrait which, in the general case of generic systems, consists of coexisting regular regions of ordered and stable motions and chaotic regions with positive Lyapunov exponents. It turns out that these structures are clearly revealed in precise correspondence with the classical counterpart if we study the properties of quantal eigenstates in the quantum phase space, such as those defined by the Wigner or Husimi functions. Moreover, the statistical properties of the energy spectra (and of other observables) behave in the semiclassical limit (of sufficiently short wavelengths), in correspondence with their classical counterparts. For classically integrable and regular systems, the statistics are Poissonian, while in the fully chaotic systems, the statistics of random matrix theories apply. These are universality classes, which do not involve any free parameters. In the mixed, (or generic) case, things naturally become more complicated, but we have theories to describe them. Furthermore, the statistical properties of the eigenfunctions (wave functions) are connected with the properties of the corresponding classical system.

In the strict semiclassical limit of a sufficiently small Planck constant, the Wigner or Husimi functions condense uniformly on the classical invariant regions, being either regular or chaotic, according to the Principle of Uniform Semiclassical Condensation (PUSC). For this, we have recently obtained quantitative phenomenological evidence, showing that the fraction of mixed states decays as a power law in the semiclassical limit, leaving behind only purely regular and purely chaotic eigenstates. However, before reaching the ultimate semiclassical limit, the chaotic Wigner functions may be localized in the quantum phase space, rather than occupying the entire classically available chaotic region. This phenomenon is the stationary aspect of dynamical or quantum localization, which is one of the most prominent phenomena in quantum chaos, and was discovered by Giulio Casati, Boris Chirikov, and Felix Izrailev in the late 1970s. The localization appears if the classical transport time is longer than the Heisenberg time. In the time-dependent domain, one observes the quantum diffusion typically only up to the Heisenberg time, which then stops due to the destructive interference. This phenomenon was originally studied in the quantum kicked rotator, both theoretically and numerically, which was introduced by Casati, Chirikov, and Izrailev as the main paradigm of quantum chaos, followed by analogous analyses in many other model and real physical systems, and stays an important subject of research to the present.

The Schrödinger equation for the wave function is just one of the many wave equations in the sense of mathematical physics that are linear and describe the propagation of waves. In the short wavelength approximation, the rays of the waves provide the leading order approximation of the wave dynamics. For example, Gaussian ray optics is the leading order approximation to the solutions of the Maxwell equations. The classical dynamics of orbits are the dynamics of rays of the quantum waves. It is clear that all observations of quantum chaos apply equally well to other wave equations in a variety of wave systems. These include electromagnetic, acoustic, elastic, surface water, seismic, gravitational, and other waves. Therefore, it would be more appropriate to talk about *wave chaos* than quantum chaos. However, the terminology of quantum chaos has been well established so far, but one must be aware of the broad subject of wave chaos that is encompassed by this research field.

Quantum chaos has experienced a noticeable decline in general interest and attention during a period of approximately several years around 2010, but became one of the top subjects in physics during the past few years once again, as it finds applications in solid state physics, fluid dynamics, molecular and atomic physics, quantum field theories, high energy physics, string theories, and even in cosmology. In addition to the theoretical investigations, there have been numerous experimental studies in many branches of physics, most notably the microwave experiments performed the more than three decades by Hans-Jürgen Stöckmann in Marburg, and also by Achim Richter in Darmstadt. There is hardly any important phenomenon or effect in quantum chaos which has not been experimentally studied by Stöckmann’s group. Furthermore, it should be noted for the new researchers in quantum chaos that the books by Stöckmann and by Haake offer the best introduction and deeper studies of quantum chaos.

Thus, the reason and motivation for organizing and editing this Special Issue of *Entropy*, titled “Quantum Chaos”, has a very solid background. As the reader can see, we have collated 36 original papers and two review papers, totaling 626 pages. Among the authors, we have many pioneers and leaders in quantum chaos, as well as some excellent researchers in the younger generation. They cover almost all important issues and aspects of quantum chaos and its developments in the last few years, covering the wide range of wave chaos in the sense described above.

Finally, I would like to stress the important reason to mark the 80th birthday of Professor Giulio Casati on this occasion, one of the most important pioneers of quantum chaos. Professor Giulio Casati has had an admirable impact on physics in a very broad sense, especially in classical and quantum chaos, where his work over more than five decades has laid down the foundations, but also in many other areas of theoretical physics and applications. Moreover, he has been, and still is, an active organizer of science, both in creating and leading elite academic and research institutions, as well as in organizing many world-top level conferences, workshops, and schools. In particular, he has been supporting young researchers all over the world in an important way. In recognition of his creative and influential life opus, and of his plentiful contributions for the good of our scientific community, this Special Issue is dedicated to him on the occasion of his 80th birthday (9 December 2022).

